# Supplementary material for: Self-Adaptive Teacher-Student framework for colon polyp segmentation from unannotated private data with public annotated datasets
Source: PLoS One. 2024 Aug 28;19(8):e0307777. doi: 10.1371/journal.pone.0307777 (PMC11356409; doi:10.1371/journal.pone.0307777)
Supplement: Attachment — Submitted filename: Response.pdf [file pone.0307777.s001.pdf]

Dear Dr. Kazunori Nagasaka,

We sincerely thank you for accepting our submission for publication with major revisions, and for giving us the opportunity to improve and resubmit our manuscript. The manuscript ID is PONE-D-24-03994. We have carefully studied each of the comments, conducted point-to-point responses, and revised the manuscript by considering all the suggestions and comments made by the reviewers.

We thank all the reviewers for appreciating our work and for their constructive suggestions.

- We would like to thank reviewer 1 for noting that, *"This paper proposed a self-adaptive teacher-student framework for polyp segmentation, to bridge the gap between the annotated datasets and the unannotated datasets. The paper is well-structured*".
- We thank reviewer 2 for praising our method "achieving satisfactory results".
- We thank reviewer 3 for considering that "*The authors address a relevant task in colon polyp segmentation on unlabeled data. It is very intuitive to leverage existing labeled data to generate pseudo-labels for unlabeled samples. A simple and effective approach is introduced to measure distribution shifts between datasets based on generative modeling of data. Experiments are quite extensive i.e., leveraging three public datasets provides evidence to support the claims.*"

In this version, we continue to improve this paper while at the same time maintaining the merits mentioned by all the reviewers.

Please find enclosed an e-copy of the revised manuscript for further consideration and a detailed reply to all the reviewers' comments and questions. For yours and the reviewers' convenience, our responses shown in purple are prepared as a point-by-point response to each of the issues raised by the reviewers. Changes to the manuscript are highlighted in red.

We hope that you and the reviewers find the major revision acceptable, and we look forward to hearing from you the final decision on our submission.

Sincerely yours,
Dr. Fu Dai (on behalf of all the co-authors)

==

Major revisions we made include:

- We revised the manuscript thoroughly to make it concise and clearer, as well as added more details to make it comprehensive, which including:

  1) We added the explanation about teacher-student framework in medical image segmentation to clarify our method name.
  2) We added the details about the three public datasets to demonstrate our dataset is appropriate.
  3) We added more explanation in each figure's caption to make the figures easier to follow.
  4) We added more details about our uncertainty-based weight to avoid future confusion.

5) We added more details about the foreground and background attention to make the method clearer.

– We implemented six experiments with corresponding discussion to make the experiments more comprehensive, which includes:

1) We added four existing methods into our comparison to make the experiment more comprehensive.

2) We added an ablation study to demonstrate the necessity of our Identify Transformer Block and Refine Transformer Block.

3) We employed fewer teacher networks and fewer public datasets to validate the effectiveness of teacher networks.

– We revised Fig.4 and Fig.6 by adding two complex examples in Fig. 4, and deepening the segmentation mask color in Fig.4 and Fig.6.

All the comments and suggestions raised by the reviewers were carefully considered. Below are our itemized responses to all the points raised by the reviewers.

==

Comments from reviewer 1:

This paper proposed a self-adaptive teacher-student framework for polyp segmentation, to bridge the gap between the annotated datasets and the unannotated datasets. The paper is well-structured but still need further improvement.

Response: We sincerely thank you for appraising that "the paper is well-structured". In this version, according to your following constructive comments and suggestions, we made clarification about our dataset size to avoiding mis-understanding. We also compared our proposed model with three recent approaches proposed in 2023 and 2024 to make the experiments more comprehensive.

Comment#1: The major problem of this paper is the experiment on the private dataset. Among the public datasets, CVC-ClinicDB has 612 cases, CVC-ColonDB has 380 cases and Kvasir has 1000 cases. However, the private dataset only has 15 cases and only 3 cases were used for model testing. Since this paper aims to solve the data distribution gap between the well-annotated public datasets and the unannotated private dataset. I am afraid such a small-scale cannot cover the heterogeneity of the polyps. How to select the sample to avoid sample selection bias? Authors should carefully response this comment, otherwise I may not be able to accept this paper due to the unconvincing experimental results.

Response#1: We sincerely thank you for the thorough review. We would like to clarify the misleading regarding the dataset sizes and address your concerns in detail.

– **Our data scale is comparable with the scale of public datasets.**

The numbers about public datasets (612, 380, and 1000) refer to the number of images (2D) collected from the cases (video sequences) instead of the number of cases. Specifically, CVC-ClinicDB[1] was collected from **23 cases**, leading to **612 images**; CVC-ColonDB[2] was

collected from **13 cases**, leading to **380 images**. The case number of Kvasir is not clearly defined in the original paper[3], but here 1000 still represents the image number rather than case number.

Our private dataset was collected from **15 cases**, leading to **1412 images** (3 cases containing 202 images were for testing), which is comparable compared with the public datasets. More specifically, our case number is bigger than CVC-ColonDB's case number, and our image number is larger than all three public datasets' image numbers.

- **Sample selection bias is avoided by random selection and the heterogeneity within each case.**

To avoid the bias in sample selection, we randomly selected 3 cases with their 202 images as the testing set. The random selection method is a commonly used statistical method that aims to reduce bias in the sample selection process so that the test set can more fairly reflect the distribution of the entire data set.

The images contained within each case itself are heterogeneous, which also improves the overall data heterogeneity. This is not only because each case usually has multiple polyps, but the images cover different angles and different lighting conditions for different numbers and types of polyps. This heterogeneity in each case ensures that our dataset has broad coverage in terms of type diversity and complexity, providing rich and challenging samples for model learning.

- **We acknowledge the data size limitation and will expand our data size in future work.**

We recognize the importance of expanding our dataset to further validate our model's performance across a wider array of clinical scenarios and polyp heterogeneity. Future efforts will focus on broadening our data collection to include more cases, thereby enhancing the generalizability and applicability of our findings.

Manuscript changes – We have clarified the description about data sizes to avoid any misunderstanding, please see page 10, lines 299-306. We also have added the discussion about our data size and future work, please see page 19, lines 506-509.

[1] Bernal, Jorge, et al. "WM-DOVA maps for accurate polyp highlighting in colonoscopy: Validation vs. saliency maps from physicians." Computerized medical imaging and graphics 43 (2015): 99-111.

[2] Vázquez, David, et al. "A benchmark for endoluminal scene segmentation of colonoscopy images." Journal of healthcare engineering 2017 (2017).

[3] Jha, Debesh, et al. "Kvasir-seg: A segmented polyp dataset." MultiMedia Modeling: 26th International Conference, MMM 2020, Daejeon, South Korea, January 5–8, 2020, Proceedings, Part II 26. Springer International Publishing, 2020.

Comment#2: In Fig. 6, the results from different methods seems have no different. In case 2, I also wonder whether authors show the same frame? Because the image content of the highlighted areas in different approaches are obvious different. But the segmentation results seem all the same. I guess the red boundaries might be the GT, then where are the segmentation results? Can authors explain this?

Response#2: Thank you sincerely for such a careful review. We have made point-to-point clarification as follows.

- **In Fig. 6, the results from different methods have differences.**
  As the zoomed image for each case in Fig. 6 shows, the result from different methods has differences. For example, in case 1, other segmentation networks, such as Mob PNet and UNet, fail to identify the polyp and output totally wrong results. In comparison, our method successfully identifies and segments the polyp. We have deepened the mask color to make the segmentation difference more noticeable.
- **In case 2, the frames are the same.**
  We double-checked the frames in case2 to ensure that they are the same.
- **The red boundaries are the GT, the blue masks are the segmentation results.**
  We employed red boundaries to illustrate the ground-truth of each polyp, and blue masks to illustrate the segmentation results. Particularly, we built blue masks according to the segmentation results and overlayed them on the polyp images. We realized that there was color distortion caused by the overlap of the mask color and the original polyp colors, which resulted in the segmentation results are not clear. Therefore, we have deepened the mask color to make the segmentation more noticeable in the revised version.

Manuscript changes – We have deepened the mask color to make the difference more noticeable. Please see page 15.

Comment#3: Why two proposed modules UDFusion and GANet are not introduced in abstract?

Response#3: Thank you sincerely for raising the good point. We have added the introduction about the two proposed modules UDFusion and GANet in the abstract as follows:

To enhance the reliability of the pseudo-labels from the teacher networks, the SATS includes a newly proposed Uncertainty and Distance Fusion (UDFusion) strategy. UDFusion dynamically adjusts the pseudo-label weights based on a novel reconstruction similarity measure, innovatively bridging the gap between private and public data distributions. To ensure accurate identification and segmentation of colon polyps, the SATS also incorporates a Granular Attention Network (GANet) architecture for both teacher and student networks. GANet first identifies polyps roughly from a global perspective by encoding long-range anatomical dependencies and then refines this identification to remove false-positive areas through multi-scale background-foreground attention.

Comment#4: The captions of figures should deliver more information to help readers better understand the technical novelties, especially Fig. 2 and Fig. 3.

Response#4: We sincerely thank you for the helpful advice. We have revised the captions of all figures to deliver more information to help readers better understand the technical novelties. For instance, the captions of Fig. 2 and Fig. 3 are revised as follows:

– Fig 2. Our SATS integrated the newly proposed UDFusion and GANet into a teacher-student protocol to achieve the unlabeled colon polyp segmentation by leveraging the labeled public datasets. Particularly, The GANet is employed to build multiple teacher modules and one student module. The teacher modules are well-trained with public datasets separately, thereby producing multiple pseudo-labels for training the student module on private data. The UDFuion evaluates the distribution between each private image and the public datasets to fuse the multiple pseudo-labels and thus outputs a reliable pseudo-label for the student modules. Therefore, the student module is able to segment private images after training without data annotation.

– Fig 3. The architecture of the proposed GANet. The GANet firstly employs ResBlocks to build the encoder and thus extract features from the input image. Then, the GANet employs the Identify Transformer Block and the Refine Transformer Blocks to build the decoder, thereby segmenting the polyps progressively. Particularly, the Identify Transformer is constructed with a channel-attention module and a spatial-attention module to predict the rough location of the polyps from a global perspective. The Refine Transformer Blocks are constructed with foreground attention and background attention under the guidance of the Identify Transformer Block, thereby predicting more accurate segmentation masks in ambiguous areas.

Comment#5: Besides SAM, authors only compared their proposed model with four competitors proposed in 2022. I suggest comparing more recent approaches proposed in 2023.
Response#5: We sincerely thank you for the good point. We have added two approaches (UniMatch[5], AugSeg[6]) proposed in 2023 and one approach (UDA-Former[4]) proposed in 2024 suggested by Reviewer #2 and Reviewer #3.

As shown in Fig. 1, our method outperforms the three methods proposed in 2023 and 2024. In the case 1, the polyp is difficult to be identified due to the complex textures of normal tissues. In this case, the UniMatch predicts a large area as the false-positive. The UDA-Former falsely identifies the normal tissues as polyp. The AugSeg also predicts some normal tissues as the polyp. In comparison, our method achieves the highest overlap rate between the prediction and the ground-truth.

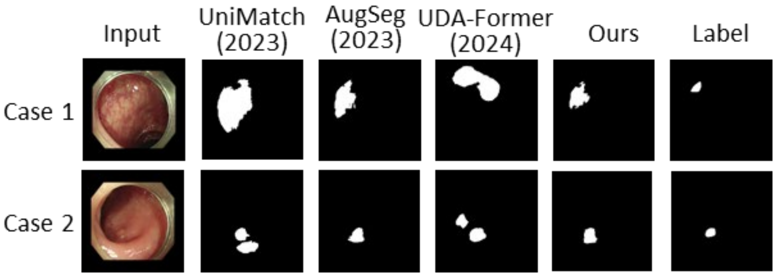

Figure 1 Comparison between methods proposed in 2023/24 and our method.

Manuscript changes – We have added the above three comparison methods into our experimental section. Please see Fig. 4 and Tab. 1.

[4] Haiqiang Liu, Meibao Yao*, Xueming Xiao, Bo Zheng, Hutao Cui. MarsScapes and UDAFormer: a Panorama Dataset and a Transformer-based Unsupervised Domain Adaptation Framework for Martian Terrain Segmentation[J]. IEEE Transactions on Geoscience and Remote Sensing, 2024, vol. 62, pp. 1-17, Art no. 4600117.

[5] Yang et al. Revisiting Weak-to-Strong Consistency in Semi-Supervised Semantic Segmentation. CVPR 23.

[6] Zhao et al. Augmentation Matters: A Simple-yet-Effective Approach to Semi-supervised Semantic Segmentation. CVPR 23.

Comment#6: English should be improved.
For example in abstract: “builds pseudo labels” should be “generates pseudo labels”
“innovatively bridging the gap” “innovatively” should be removed.
“This strategy mainly dynamically” “mainly” should be removed.

Response#6: We sincerely thank you for pointing out these grammar issues. We have invited a native English speaker to improve the English. For instance, the sentence “builds pseudo labels” has been revised as “generates pseudo labels”. The sentences “innovatively bridging the gap” and “This strategy mainly dynamically” have been revised as “bridging the gap” and “This strategy dynamically”, respectively.

Comments from reviewer 2:

This paper introduces the Self-Adaptive Teacher-Student (SATS) framework, an unsupervised approach for colon polyp segmentation based on a teacher-student architecture. The SATS method comprises two primary phases. In the initial phase, pseudo-labels are generated by training three teacher networks on three existing public datasets. Using unannotated private data as input, the Uncertainty and Distance Fusion (UDFusion) method processes the outputs of teacher networks to obtain adaptive pseudo-labels. The second phase involves training a student network. Pseudo-labels generated in the prior phase are utilized to train a Grainy Attention Network (GANet), ensuring accurate identification and segmentation of colon polyps. Despite achieving satisfactory results, the paper still faces challenges in terms of method description and experimental aspects:

Response: We sincerely thank you for assessing that “achieving satisfactory results”. In this version, according to your following constructive suggestions, we improved this paper to avoid the confusion or concerns for future readers.

Comment#1: Is the term "Teacher-Student" framework appropriate for this paper? Because this study involves a two-step unsupervised semantic segmentation process. Initially, three networks are trained using three public datasets, and pseudo-labels are generated using these trained networks. Subsequently, a separate network is trained using these pseudo-labels. The so-called teacher-student networks do not participate in the same framework for training and prediction. Moreover, they share identical structures.

Response#1: Thank you sincerely for raising this concern. We have made clarifying that “Teacher-Student” framework is appropriate for this paper. We also have added the explanation about “Teacher-Student” framework in the manuscript to avoid further confusion.

We recognize that “Teacher-Student” has traditionally been primarily associated with knowledge distillation processes where a teacher model directly guides the learning of a student model. However, in recent years, the "teacher-student" concept has been widely used in a variety of learning frameworks, not limited to direct knowledge transfer. To name a few, Yu et al.[7] built a “teacher model” to generate pseudo-labels and thus trained a “student model”, thereby achieving semi-supervised medical segmentation. Zhang et al.[8] built a “teacher-student” network to achieve non-enhanced tumor segmentation with the assistance of enhanced images, where the teacher network and the student network share identical structures. Chen et al.[9] employed “teacher-student” framework to learn rotation-invariant features for segmentation.

In our study, we exploited the core idea of the "teacher-student" concept - that is, using the output of advanced models to guide the training process of other basic models. Specifically, we achieve label-free training by generating pseudo-labels from a model trained on three different public datasets (as the “teacher”) and then using these pseudo-labels to train another independent model (the “student”). This process embodies an indirect knowledge transfer and guidance, consistent with the broad definition of the “teacher-student” concept. We choose to

use the term "teacher-student network" because we hope to emphasize the idea of knowledge transfer and learning guidance through pseudo-labels in an unsupervised learning environment.

We understand the reviewer's concerns about the use of terminology and further clarify in the paper the connections and differences between our approach and traditional "teacher-student" networks. Therefore, we have added the broad application of the “teacher-student” concept in related work to demonstrate the rationality of our terminology choice. We believe that this indirect learning guidance method based on pseudo-labels, although different in technical implementation from traditional knowledge distillation technology, still follows the core spirit and goals of the "teacher-student" model.

Manuscript changes – We have added the introduction about “teacher-student” framework in the related section to clarify the difference between our method and the traditional knowledge-distillation methods, please see pages 4-5, lines 133-149.

[7] Yu, Lequan, et al. "Uncertainty-aware self-ensembling model for semi-supervised 3D left atrium segmentation." Medical image computing and computer assisted intervention–MICCAI 2019: 22nd international conference, Shenzhen, China, October 13–17, 2019, proceedings, part II 22. Springer International Publishing, 2019.

[8] Zhang, Dong, et al. "Weakly-supervised teacher-student network for liver tumor segmentation from non-enhanced images." Medical Image Analysis 70 (2021): 102005.

[9] Chen, Han, et al. "A teacher–student framework with Fourier Transform augmentation for COVID-19 infection segmentation in CT images." Biomedical Signal Processing and Control 79 (2023): 104250.

Comment#2: It appears that Figure 1(b) lacks a specific explanation of the feature distribution plot. Could you please clarify the meanings of the horizontal and vertical axes?

Response#2: We sincerely thank you for raising this good point. We have added the specific explanation of the feature distribution plot to make the figure clearer.

Figure 1(b) shows the feature distribution of different data sets in low-dimensional space by using the UMAP algorithm[10] to reduce the dimensionality and visualize the data features. UMAP is a popular nonlinear dimensionality reduction technique that aims to maintain the local and global structure of high-dimensional data and reveal the intrinsic structure of the data in the reduced space.

The horizontal axis (X-axis) and the vertical axis (Y-axis) in Figure 1(b) represent the two main dimensions after dimensionality reduction by the UMAP algorithm. These two axes themselves do not correspond to specific features in the original feature space, but are new coordinate systems generated by UMAP through its optimization process based on the geometric structure of the data distribution. Therefore, these two dimensions are abstract and mainly used for visualization purposes, allowing us to observe and analyze the distribution of data in a low-dimensional space.

Manuscript changes – We have added the specific explanation of the feature distribution plot in the caption of Figure 1 to make the figure easy to understand, please see page 2.

[10] McInnes, Leland, John Healy, and James Melville. "Umap: Uniform manifold approximation and projection for dimension reduction." arXiv preprint arXiv:1802.03426 (2018).

Comment#3: Is the notation 'wivi(p, q)' on line 158 written incorrectly and should it be 'wi(p, q)'? On line 173, is the description 'of at (p, q)' correct?

Response#3: We sincerely thank you for the careful review and pointing these typos out. We have corrected the notation on line 158 and the description on line 173. We also have double checked other notations and descriptions to ensure that they are all correct.

- On line 158, the notation should be $\frac{u^i+v^i(p,q)}{2}$, where u^i is the distribution weight between each image and the i-th public dataset. Namely, u^i is an image-wise weight. $v^i(p,q)$ is the uncertainty wight by the i-th teacher module at pixel (p,q), indicating $v^i(p,q)$ is a pixel-wise weight.
- On line 173, the description should be “at (p,q)”.

Manuscript changes – We have corrected the above two typos and made a careful proof-reading to ensure there is no more errors.

Comment#4: The Uncertainty and Distance Fusion (UDFusion) method calculates weights on a per-pixel basis, lacking consideration for the correlation between pixels. Does this lead to the pseudo-label images losing contextual information of spatial pixels? It is hoped that the authors can provide an explanation for this issue.

Response#4: We sincerely thank you for raising this good point. The UDFusion method calculates weights on a per-image basis and a per-pixel basis, which leads to the pseudo-label images reserving contextual information of spatial pixels.

More specifically, as we have mentioned in the response for Comment#3, the weight by UDFuion is defined as $\omega^i(p,q) = \frac{u^i+v^i(p,q)}{2}$. Namely, the UDFusion method takes the image-wise distribution weight u^i and the pixel-wise uncertainty weight $v^i(p,q)$ into consideration. Particularly, u^i is the distribution weight between each image and the i-th public dataset, which evaluates the reliability of each pseudo label through the contextual information. $v^i(p,q)$ is the uncertainty wight by the i-th teacher module at pixel (p,q), which evaluates the reliability of each pseudo label through the pixel information. By integrating the distribution weight and the uncertainty weight, the UDFusion is able to reserve both contextual information of spatial pixels and local information at pixel.

Manuscript changes – We have added the explanation about how the UDFusion method reserves contextual information to avoid future confusion. Please see page 6, lines 176-180.

Comment#5: Formulas 6 and 7 provide limited descriptions of the uncertainty weight. Can there be an appropriate addition to the explanation of the interpretability of the uncertainty weight and the reasons behind its design?

Response#5: We sincerely thank you for this constructive suggestion to make our method clearer. We have added the explanation of the interpretability of the uncertainty weight and the reasons behind its design.

- **Interpretability of the uncertainty weight.**

$$U^i(p,q) = -\hat{y}^i(p,q) \times \log\left(\hat{y}^i(p,q)\right)$$

This formula quantifies the uncertainty of the i-th teacher module's prediction $\hat{y}$ at every pixel (p,q) based on its entropy $\hat{y} \times \log(\hat{y})$. Entropy is a commonly employed method to effectively measure how scattered or concentrated the model's predictions are, with higher entropy indicating less certainty[11]. More specifically, if $\hat{y}$ is close to 1 or 0, the entropy will be very low, which indicates the model is certain to classify the pixel as background or foreground. Otherwise, if $\hat{y}$ is close to 0.5, the entropy will be very high, which indicates the model is uncertain to classify the pixel as background or foreground. Entropy-based uncertainty is particularly valuable in medical imaging because it helps identify regions of an image where the model cannot classify them, suggesting possible ambiguities in anatomical boundaries or pathological features. For example, in semi-supervised left atrium segmentation, entropy can identify the areas of an atrium image where the model is less certain, leading to a small loss weight in these areas[4].

$$v^i(p,q) = \text{softmax}\left(U^i\right) = \frac{e^{-U^i(p,q)}}{\sum_{j=1}^{k} e^{-U^j(p,q)}}$$

This formula normalizes the uncertainty values across all teacher modules using a softmax function. The transformation $\frac{e^{-U^i(p,q)}}{\sum_{j=1}^{k} e^{-U^j(p,q)}}$ ensures that a lower uncertainty leads to a higher weight during generating the ultimate reliable pseudo-label.

- **The reasons behind its design.**

Uncertainty of the segmentation can indirectly reflect the distribution difference between the public datasets and the input private images. In multiple prediction results by teacher modules, a higher uncertainty value indicates the teacher module is ambiguous at this pixel, which means features at this pixel is out of the distributions during training on the public dataset. Similarly, a lower uncertainty value indicates the teacher module is confident at this point, which means features at this pixel is in the distribution during training on the public dataset. Through the different uncertainty values, we can identify the distribution difference between the public datasets and the private input image. Consequently, we can construct a more reliable pseudo label by allocating a higher weight to the prediction with a low uncertainty value.

Manuscript changes – We have added the explanation of the interpretability of the uncertainty weight and the reasons behind its design. Please see page 7 lines 196-201.

[11] Shannon, Claude Elwood. "A mathematical theory of communication." The Bell system technical journal 27.3 (1948): 379-423.

Comment#6: The correspondence between the modules in the architecture of Figure 3 and the described content in the text is not clearly illustrated. Additionally, the explanation in the text regarding the 'foreground attention' and 'background attention' of the Refine Transformer Block, and how they continuously correct mispredicted areas, is not sufficiently thorough.

Response#6: We sincerely thank you for these good points to make our presentation clearer. We have revised the content and added subheadings to each module in the text to make its correspondence with the modules in Figure 3 clearly illustrated. We also added the explanation in the text regarding the 'foreground attention' and 'background attention' of the Refine Transformer Block, and how they continuously correct mis-predicted areas. Please see below for details.

– **The content in the text corresponding to the modules in Figure 3.**

As shown by the left of Fig. 3, GANet firstly employs ResBlocks to downsample the input images and thus compress redundant features. Then, GANet employs the newly-proposed Identify Transformer Block and Refine Transformer Block to localize and segment polyps progressively. As illustrated by the right of Fig.3, the Identify Transformer Block learns to predict the coarse location of polyps, and the Refine Transformer Block gradually makes the prediction clearer by integrating the coarse prediction by previous block and the features from ResBlocks.

– **The explanation regarding the 'foreground attention' and 'background attention' of the Refine Transformer Block.**

The foreground attention $Attn_{sp}^{fg}$ employs the self-attention mechanism to refine the foreground feature filtered with the coarse prediction by the previous decoder block. Namely,

$$\begin{cases} z_d^{fg} = z_d P_{d+1} \\ z_d^{fg} = \mathrm{Attn}_{sp}^{fg}\left(z_d^{fg}\right) \end{cases}$$

Where P_{d+1} is the polyp mask predicted by the previous decoder block, with intensity 1 as the foreground and intensity 0 as the background; z_d is the feature from ResBlock at the same level. Similarly, we can get the process that the background attention $Attn_{sp}^{bg}$ refines the background features:

$$\begin{cases} z_d^{bg} = z_d(1 - P_{d+1}) \\ z_d^{bg} = \mathrm{Attn}_{sp}^{bg}\left(z_d^{bg}\right) \end{cases}$$

Because both the foreground attention and background attention are based on the self-attention mechanism, we formular them as $\mathrm{Attn}_{sp}^{f/bg}$ for simplicity. Similar to the spatial-

attention module, the process of $\mathrm{Attn}_{sp}^{f/bg}$ refining the foreground/background features $z_d^{f/bg}$ can be formularized as:

$$\begin{cases} Q_{f/bg} = \omega_{f/bg}^{q} \otimes z_d^{f/bg} \\ K_{f/bg} = \omega_{f/bg}^{k} \otimes z_d^{f/bg} \\ V_{f/bg} = \omega_{f/bg}^{v} \otimes z_d^{f/bg} \\ A_{f/bg} = \mathrm{softmax}\left(\frac{Q_{f/bg} \times K_{f/bg}^{T}}{\sqrt{c}}\right) \\ z_d^{f/bg} = A_{f/bg} \times V_{f/bg} \end{cases}$$

Where ω is the convolutional kernel to be learned.

– **how they continuously correct mispredicted areas.**

The foreground attention and background attention correct mis-predicted areas by three actions:

1) divide the encoded features from ResBlocks into foreground features z_d^{fg} and background features z_d^{bg} by multiplying the features z_d with foreground mask P_{d+1} and background mask 1-P_{d+1}, which enables the foreground and background features can be processed specifically.

2) Remove the wrong foreground features z_d^{fg} from the coarse features z_d from previous decoder block and add the wrong background features (or the correct foreground features) z_d^{bg}, which make the foreground features for mask prediction cleaner.

3) Loss function between the predicted mask and the ground truth enables the foreground attention and background attention to captures wrong features in both foreground and background regions.

Manuscript changes – We have added the content and subheadings to each module in the text to make its correspondence with the modules in Figure 3 clearly illustrated, please see pages 7-9, line 212 and 243. We also added the explanation in the text regarding the 'foreground attention' and 'background attention' of the Refine Transformer Block, and how they continuously correct mis-predicted areas. Please see page 10, line 267-284, lines 291-295.

Comment#7: In Formula 11, why is 'zfg' subtracted from 'zd+1' and added to 'zbg'? Both 'zfg' and 'zbg' are calculated from the same two feature maps, with the only difference being the sign choice for Pd+1. What is the significance of the dual branches?

Response#7: We sincerely thank you for raising this good point.

– **Why is $\boldsymbol{z_{fg}}$ subtracted from $\boldsymbol{z_{d+1}}$ add added to $\boldsymbol{z_{bg}}$?**

1) $\boldsymbol{z_{fg}}$ is subtracted from $\boldsymbol{z_{d+1}}$ because $\boldsymbol{z_{d+1}}$ is the coarse foreground feature used by the previous decoder block to predict the previous mask, while $\boldsymbol{z_{fg}}$ is the wrong

foreground features. z_{fg} is subtracted from z_{d+1} is to remove the wrong foreground features from the previous coarse foreground features.

2) and added to z_{bg} is because z_{bg} is the wrong background features, i.e., z_{bg} is the useful foreground features actually. By adding z_{bg}, the foreground features are completed, thereby predicting more accurate foreground mask.
3) Although originally both z_{bg} and z_{bg} are calculated from the same two feature maps, they are refined by the 'foreground attention' and 'background attention' respectively. Namely, before subtracting and adding, z_{bg} and z_{bg} are totally different the original version calculated from the same two feature maps.

– **What is the significance of the dual branches?**

The dual branch strategy that processes foreground and background features separately enables each branch to focus more on feature extraction in its respective area. More specifically, the distinction between foreground (polyp) and background (normal tissue) is obvious. By processing them separately, the model can distinguish these regions more effectively, thereby reducing the possibility of misidentification. More importantly, by treating the foreground and background separately, the feature extraction process for foreground or background can be tuned more precisely. Namely, it allows the model to be more focused and efficient in processing their respective regions, especially when subtle differences in feature expression need to be distinguished.

Manuscript changes – We have added the reason that why 'zfg' is subtracted from 'zd+1' and added to 'zbg' to make the method clearer, please see page 10, line 267-276. We have also added significance of the dual branches to make the method comprehensive, please see page 10, lines 276-284.

Comment#8: In Figure 3, there is an input 'Od+1' in the 'zfg' branch, but there is no explanation for the 'Od+1' input from Figure 3 in Formula 11.

Response#8: We sincerely thank you raising up this question. In the manuscript, we explained the 'Od+1' input from Figure 3, "Od+1 are the up-sampled features from the previous block (Refine Transformer Block or Identify Transformer Block)". Please see page 9, lines 246-248.

Comment #9: Most of the visualization examples in Figure 4 are cases where the polyp shapes have smooth and regular boundaries. How does the SATS method perform in cases with complex boundaries? In Figure 4, for cases 1 and 3, the segmentation edges obtained by the method in this paper are not clear and appear mosaic-like. What could be the reason for this?

Response#9: We sincerely thank you for this careful suggestion.

We have added two examples with complex boundaries to show the performance of the SATS. As demonstrated in Fig. 2 (Fig. 4 in manuscript), compared with existing methods, our SATS achieves the highest overlap rate between the prediction and the ground-truth. For instance, in the case 1, the polyp is difficult to be identified due to the complex textures of normal tissues. In this case, most of existing methods fail to identify the polyp, such as the C2F,

or predict a large area as the false-positive, such as the DAN and the CAM. In comparison, our method identifies the polyp correctly and predicts the lowest false-positive area.

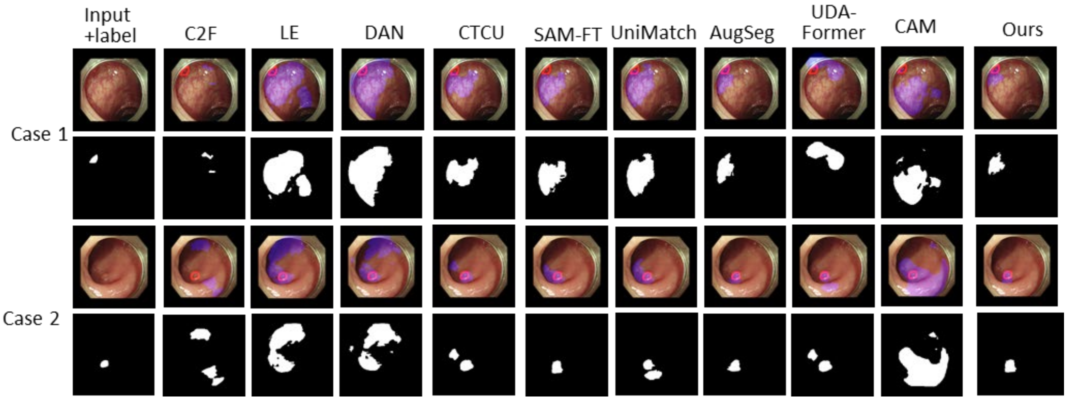

Figure 2 Comparison between our method and existing methods.

The reasons for the segmentation edges obtained by our method in this paper are not clear and appear mosaic-like can be attributed to the following two points:

a) The prediction from teacher networks in the private data is not accurate, which causes the student network to be trained with unclear supervision. There is a distribution difference between public datasets and our private dataset. This difference leads to differences between pseudo and real labels, which causes the student network to be unstable during training and testing.

b) The prediction results from multiple teacher networks in the private data are various, which causes the fused pseudo-label is not clear and appear mosaic-like. There are distribution biases among public datasets, which causes the teacher networks trained with different public datasets to predict different results. After fusing these different results, the ultimate pseudo-label can be mosaic-like.

Manuscript changes – We have added two examples with complex boundaries to show the performance of the SATS. Please see Fig. 4 on page 12 for more details.

Comment #10: In Table 1, were the other comparison experiments conducted using the same networks as those proposed in this paper, and were they also trained using the three public datasets? How was fairness ensured in the comparison? How do the parameter count and complexity of the compared methods compare? It is recommended to include some networks with similar attention structures in the comparison experiments for more convincing results.
For example:

1)Yu C, Xiao B, Gao C, et al. Lite-hrnet: A lightweight high-resolution network[C]//Proceedings of the IEEE/CVF conference on computer vision and pattern recognition. 2021: 10440-10450.

2)Haiqiang Liu, Meibao Yao*, Xueming Xiao, Bo Zheng, Hutao Cui. MarsScapes and UDAFormer: a Panorama Dataset and a Transformer-based Unsupervised Domain Adaptation Framework for Martian Terrain Segmentation[J]. IEEE Transactions on Geoscience and Remote Sensing, 2024, vol. 62, pp. 1-17, Art no. 4600117, https://doi.org/10.1109/TGRS.2023.3343109.

Response#10: We sincerely thank you for these good points and the helpful suggestion.

Yes, these comparison experiments were conducted using the same networks as those proposed and the same hyper-parameters in their papers. They were also trained using the same three public datasets. To ensure the fairness in the comparison, we made sure that all methods were trained and validated with the same data, and all methods were implemented with the same hardware and software environment.

The parameter count and complexity of the compared methods were not compared in our experiments. The main reason is our research focus at this stage is to validate the effectiveness of the proposed method in segmentation. In our future work, we will reduce the complexity of our method and then compare it with existing methods.

Following your suggestions and other two reviewers' suggestions, we added UDAFormer and other three methods (AugSeg, UniMatch, and CAM) proposed after 2022 into our comparison in our revised version. As shown in Tab. 1, our method outperforms these existing methods in terms of five matrices out of seven evaluation matrices. Particularly, UDA-Former obtains an IoU of 72.79%, a Recall of 72.81%, and a HD of 10.15, which is under-performing compared to our method.

Table 1 Comparison between existing methods and our method

	IoU [%]	Dice [%]	Prec [%]	Recall [%]	Acc [%]	HD [Pixels]	MSD [Pixels]
UniMatch	71.85 ±24.33	83.7 ±24.21	90.25 ±23.78	71.86 ±24.91	98.61 ±0.06	12.67 ±8.39	5.40 ±5.34
AugSeg	73.29 ±15.22	84.55 ±10.57	**93.37 ±6.10**	73.30 ±13.25	**98.68 ±0.02**	9.65 ±2.78	4.45 ±1.21
UDA-Former	72.79 ±17.75	84.25 ±18.31	91.98 ±20.49	72.81 ±12.27	98.54 ±0.08	10.15 ±6.48	5.23 ±2.47
CAM	52.72 ±40.48	69.04 ±37.21	63.89 ±34.81	52.72 ±35.53	97.09 ±0.57	15.49 ±18.18	8.63 ±16.44
**Ours**	**76.30 ±13.06**	**86.67 ±9.76**	89.98 ±12.92	**86.00 ±16.75**	98.36 ±0.12	**7.01 ±1.09**	**3.48 ±0.49**

Manuscript changes – We have added the above comparison experiment into our manuscript. We also added the future work to include complexity evaluation. Please see page 19, lines 509-513.

Comment #11: The Ablation studies lack sufficient evidence and justification for the roles and necessity of the Identity Transformer and Refine Transformer modules.

Response#11: We sincerely thank you for this careful suggestion. We have added an ablation study to demonstrate the necessity of the Identity Transformer and Refine Transformer modules. As shown in Tab. 2, we employ the ResUNet as the baseline, and validate the improvement by the Identify Transformer Block and the Refine Transformer Block. Compared with the baseline method, the Identify Transformer Block and the Refine Transformer Block significantly improve segmentation performance in terms of the four evaluation matrices. By integrating the Identify Transformer Block and the Refine Transformer Block, our method obtains the best performance.

Table 2 Ablation study to show the necessity of the Identify Transformer block and the Refine Transformer block.

Method	Dice	Prec	Acc	MSD
Baseline (ResUNet)	49.18	56.20	95.26	57.30
Baseline w/ Identify Transformer	75.70	79.58	98.68	6.03
Baseline w/ Refine Transformer	78.88	79.26	98.87	7.29
Identify & Refine Transformers (Ours)	86.67	89.98	98.36	3.48

Manuscript changes – We have added the above ablation study into our experimental section to make the manuscript comprehensive. Please see page 14, lines 407-416 for details.

Comments from reviewer 3:

The authors propose a student-teacher framework for semi-supervised segmentation of colon polyps on unlabeled video data by leveraging dynamic pseudo-label generation across teacher networks trained on multiple datasets. To measure distribution discrepancy between public labeled and private unlabeled datasets, reconstruction similarity is used a metric from public data trained auto-encoders. To enhance segmentation prediction from pseudo labels, the authors introduce refinement modules to suppress false predictions and incorporate multi-scale information to retain anatomical details. Evaluation using three public datasets for pseudo-label generation by training on a single private unlabeled dataset reveal significant gains over prior art, validating the intuition behind the designs.

- The authors address a relevant task in colon polyp segmentation on unlabeled data. It is very intuitive to leverage existing labeled data to generate pseudo-labels for unlabeled samples.
- An simple and effective approach is introduced to measure distribution shifts between datasets based on generative modeling of data. Given the estimated shifts, the author leverage it as prior to enhance the learning of a student network.
- Experiments are quite extensive i.e., leveraging three public datasets provides evidence to support the claims.

Response: We sincerely thank you for appraising that "The authors address a relevant task in colon polyp segmentation on unlabeled data", "A simple and effective approach is introduced to measure distribution shifts between datasets based on generative modeling of data". In the revised manuscript, we have followed all your comments to improve our methodology.

Comment#1: While I appreciate the experimental setting, It is unclear if the compared methods were trained on the full public dataset (i.e., CVC-ClinicDB + CVC-ColonDB + Kvasir) or a select dataset. Since the proposed approach uses all three, it is important to explicitly highlight this point in the paper.

Response#1: We sincerely thank you for pointing this out. The compared methods were trained on the full public datasets to ensure the comparison setting is fair.

Manuscript changes – We have added the explanation about the experimental setting to make the presentation comprehensive, please see page 13 lines 362-363.

Comment#2: As the proposed approach leverages multiple teachers, it is important to establish a lower-bound for the sensitivity of the approach by using fewer (≤ K) teachers/datasets. This can better contextualize the source of improvement since it is not clear if the approach will work with a single teacher. Moreover, the evaluation does not include popular semi-supervised approaches. (see references below).

- Yang et al. Revisiting Weak-to-Strong Consistency in Semi-Supervised Semantic Segmentation. CVPR 23
- Zhao et al. Augmentation Matters: A Simple-yet-Effective Approach to Semi-supervised Semantic Segmentation. CVPR 23

- Chen et al. C-CAM: Causal CAM for Weakly Supervised Semantic Segmentation on Medical Image. CVPR 22.

Response#2: Thank you for raising these good points.

- We have added experiments by using fewer teachers and fewer public datasets. As shown in Tab. 3, when there is one teacher network, the Kvasir dataset facilitates the best performance compared with the other two datasets, indicating that the distribution of the Kvasir dataset is closet to our private data. With more teacher networks being included, the performance is increasing.

Table 3 The performance with fewer teachers and fewer public dataset

Datasets			Evaluation		
ClinicDB	ColonDB	Kvasir	Dice	Recall	MSD
√			78.46	64.56	8.71
	√		78.19	64.19	7.91
		√	80.89	67.91	7.36
√	√		79.74	66.30	7.59
√		√	83.18	71.21	5.83
	√	√	85.71	75.01	5.72
√	√	√	**86.67**	**86.00**	**3.48**

- We also have included popular semi-supervised approaches in our experiments. As shown in Tab. 3, our method outperforms these three approaches in terms of 5 matrices out of 7 evaluation matrices. For instance, our method outperforms these three approaches more than 3% on the IoU, more than 2.5 pixels on the HD.

Table 4 The comparison between our method and existing methods

	IoU [%]	Dice [%]	Prec [%]	Recall [%]	Acc [%]	HD [Pixels]	MSD [Pixels]
UniMatch	71.85 ±24.33	83.7 ±24.21	90.25 ±23.78	71.86 ±24.91	98.61 ±0.06	12.67 ±8.39	5.40 ±5.34
AugSeg	73.29 ±15.22	84.55 ±10.57	**93.37 ±6.10**	73.30 ±13.25	**98.68 ±0.02**	9.65 ±2.78	4.45 ±1.21
CAM	52.72 ±40.48	69.04 ±37.21	63.89 ±34.81	52.72 ±35.53	97.09 ±0.57	15.49 ±18.18	8.63 ±16.44
**Ours**	**76.30 ±13.06**	**86.67 ±9.76**	89.98 ±12.92	**86.00 ±16.75**	98.36 ±0.12	**7.01 ±1.09**	**3.48 ±0.49**

Manuscript changes – We have added the above two experiments into our manuscript to make it more comprehensive. Please see page 13 and page 18.

Comment#3: The motivation behind using the L_tversky loss is not elaborated clearly. Additionally, the is no citation/reference to the original. More details on this objective need to be clarified.

Response#3: We sincerely thank you for raising this good point. We have elaborated L_tversky loss as following and updated our manuscript accordingly to make it comprehensive.

- **Motivation:** We employed Tversky loss because it adapts to the class imbalance problem between foreground and background in the given image, which thus has been a common loss function for medical image segmentation. Particularly, when processing colon polyp images, we face the challenge of sample imbalance, where the polyps present in small regions relative to the background normal tissue. Tversky loss through its parameters α and β provides flexibility to adjust the weights of false positives and false negatives, which allows us to tune the sensitivity of the segmentation algorithm more finely to the small foreground regions.
- **Reference:** The original Tversky loss was proposed in the 2017 work of Salehi et al. (Reference: Salehi, S. S. M., Erdogmus, D., & Gholipour, A. (2017). Tversky loss function for image segmentation using 3D fully convolutional deep networks).

Manuscript changes – We have added the motivation about Tversky loss, and the citation about this loss function in the manuscript for readers' reference, please see page 10, lines 290-295.

Comment#4: The writing and descriptions are not verbose. For instance, several instances of lose wording and unclear descriptions e.g., L380-381 in the selection experiment section. L400 should be 'Evaluation metrics'?

Response#4: We sincerely thank you for pointing out these typos and issues. We have corrected these issues. For instance, the sentences in L380-381 have been revised as "However, if the coefficient for the larger prediction is excessively large, such as 3^d, the segmentation performance will decrease. This occurs because the coefficients assigned to smaller predictions are too small, rendering these smaller predictions ineffective in enhancing the overall prediction performance." We have also conducted a thorough proofreading to ensure that the writing and descriptions are grammatically correct.
