# Supplementary material for: Self-Adaptive Teacher-Student framework for colon polyp segmentation from unannotated private data with public annotated datasets
Source: PLoS One. 2024 Aug 28;19(8):e0307777. doi: 10.1371/journal.pone.0307777 (PMC11356409; doi:10.1371/journal.pone.0307777)
Supplement: Attachment — Submitted filename: Response to Reviewers.pdf [file pone.0307777.s002.pdf]

To: Dr. Kazunori Nagasaka
Academic Editor, *PLOS ONE*

Dear Dr. Kazunori Nagasaka and reviewers:

We are very grateful for your minor revision and potentially accepting our manuscript for publication in the Journal of Plos One. We would like to thank you and all the reviewers for your very constructive comments and useful suggestions in the previous major revision, which have greatly improved our manuscript.

We have sent you a new revised version together with a letter explaining all the modifications, which contain each point raised by the reviewers, our point-by-point response, and the corresponding modifications in the manuscript. All modifications are highlighted in red in the article.

Looking forward to hearing from you about the final decision on our submission.

Sincerely Yours,
Dr. Fu Dai (on behalf of all coauthors)

==

Comments from reviewer #1:

Authors have addressed all the comments. I have no further comment. This paper is ready to be published.

RESPONSE: We want to sincerely thank you for appreciating our work. Your encouragement, positive reviews, and very helpful suggestions are critical to the success of our manuscript, and it will greatly benefit further readers.

==

Comments from reviewer #2:

Thank you to the authors for providing detailed responses and revisions to the previous concerns. Most of the issues in the paper have been addressed. However, there are still a few questions that need further clarification.

RESPONSE: We sincerely thank you for your consent to our revisions to the paper. The manuscript is much clearer to follow after a substantial revision with your previous suggestions, which will greatly benefit for further readers. We have made point-to-point response to your questions.

Comment 1. In the experimental results section, it seems that the distribution bias among the public datasets leads to unstable prediction results. Did the authors decide the selection of the three public datasets with any specific criteria in mind, or were any pre-processing methods used to enhance the stability of pseudo-label generation?

RESPONSE: We sincerely thank you for raising these questions.

- The selection of the three public datasets involves following specific criteria:

1. *Public availability and usage in the community***.** We selected three datasets, CVC-ClinicDB, CVC-ColonDB, and Kvasir, are widely recognized and used in the research community, facilitating comparison with other methods and ensuring reproducibility.

2. *Annotation quality***.** We prioritized datasets with high-quality, expert-annotated ground truth to ensure reliable training of the teacher networks, thereby preventing cumulative errors in the student networks.

- Distribution bias is addressed via our proposed UDFusion strategy, instead of pre-processing methods.

The core innovation of our method is to leverage the distribution biases among different datasets to generate pseudo-labels of varying quality. Instead of pre-processing to enhance the pseudo-label quality, we embrace these differences as they provide a rich source of information that our framework can utilize. Specifically, our UDFusion strategy dynamically adjusts the weights of the pseudo-labels generated by the teacher networks, which effectively integrates the varying levels of reliability from different datasets, improving the overall robustness and accuracy of the student network.

Manuscript changes – We have added the specific criteria about public dataset selection in our manuscript to make it more comprehensive, please see page 10, lines 302-304. We have also added the declaration that we specifically designed UDFusion strategy to utilize the pseudo-labels with different qualities instead of using any pre-processing methods to address the distribution bias, please see page 11, lines 319-323.

Comment 2. In line 176, it is mentioned that the UDFusion module uses ui to assess the reliability of pseudo-labels through contextual information. How is this contextual information represented? Is there a direct connection between formula (1) and the unstable distribution in the final visualized results?

RESPONSE: We sincerely thank you for these good questions.

- As explained in Eq. (4) and (5) in the manuscript, the contextual information for u_i is represented by the distribution distances between a private image and the

public datasets. More specifically, the UDFUsion module pretrains three encoder-decoder networks with the three public datasets. By inputting a private image into these pretrained networks and evaluating the restored image qualities, the UDFUsion module can get the distribution distance between the input private image and the three public datasets. Because the distribution distance evaluation focuses on the overall contextual information between the input image and the restored image, we formulate that the UDFusion module uses u_i to assess the reliability of pseudo-labels through contextual information.

- Yes, there is a direct connection between formula (1) and the unstable distribution in the final visualized results. Formula (1) represents the process to build pseudo-labels which are used for training the student module. Due to the limited public dataset numbers, the pseudo-labels are unstable, further causes the unstable prediction in the final visualized results.

Manuscript changes – We have added the explanation about how the contextual information is represented for u_i to make the manuscript clearer, please see page 6, lines 178-179. We also added the discussion about the connection between formula (1) and the unstable distribution in the final visualized results, please see page 19, lines 513-515.

Comment 3. In line 189, there are two colons ":". Is this a typographical error?

RESPONSE: We sincerely thank you for pointing out this typo. We have double-checked every sentence to ensure there are no typos.
